# Self-regulatory employability attributes and competency: the strengthening role of grit

**DOI:** 10.3389/fpsyg.2023.1298299

**Published:** 2023-11-28

**Authors:** Sadika Ismail, Ingrid L. Potgieter, Melinde Coetzee

**Affiliations:** ^1^Department of Human Resource Management, University of South Africa, Pretoria, South Africa; ^2^Department of Industrial and Organisational Psychology, University of South Africa, Pretoria, South Africa

**Keywords:** autonomy, career agility, consistency of interest, cultural ingenuity, self-regulation, perseverance of effort, proactive career resilience

## Abstract

**Introduction:**

This study examines grit as psychological mindsets that explain the link between self-regulatory employability attributes and perceived employability competency expectations in a sample of South African adults (*N* = 308).

**Methods:**

A quantitative, cross-sectional research design approach was used to collect primary data.

**Results:**

Results of a mediation analysis through structural equation modelling revealed grit as an important mechanism to strengthen the association between employability attributes (career agility, cultural ingenuity, proactive career resilience) and employability competency expectations (autonomy/leadership skills and personal employability qualities).

**Discussion:**

This study makes an important contribution to the role of learning and training through understanding the role of grit in enhancing prospects of employability. This study further adds to the grit literature, highlighting the role that grit plays in the contemporary employment context. Practical implications include supportive practices that strengthen individual workers’ grit when confronted with the turbulent changes of today’s work world.

## 1 Introduction

Today’s work world demands continuous upskilling and reskilling resulting from the rapid pace of technological changes which necessitates further education and training for sustained employability (Whysall et al., 2019; Suarta and Suwintana, 2021; Business Tech, 2022; Li, 2022; O'Donnell, 2022; Tran et al., 2024). Employers expect employees to be well-rounded individuals that possess competencies and broader transferable skills and attributes (employability qualities) in addition to their discipline-specific knowledge (graduateness), which will allow them to be competent, energetic and informed citizens who play a crucial role in technological advancements for value-added products and services and general business success (Tomlinson, 2012; Coetzee, 2017; Ismail, 2017; Clarke, 2018). Employability and self-development now take precedence over job security and loyalty to the organisation (Costa, 2021; Raeder, 2021).

Being aware of the competency expectations of employers may enhance individuals’ employability and may also significantly narrow the gap between the supply and demand within the labour market (Steurer et al., 2022). Individuals thus need to display career self-management behaviours such as career agility, cultural ingenuity and proactive career resilience in taking responsibility for the planning of their own careers. These three self-regulatory employability attributes inform perceptions of meeting the employability competency expectations of employers (Pham, 2021). Stated differently, when individuals’ self-regulatory employability attributes are high, they perceive that they are better able to meet employer’s employability competency expectations. Agentic (self-regulated) behaviours are exhibited in attributes and competencies, including motivational mindsets such as grit to achieve employability (Baluku et al., 2021; Del Castillo and Lopez-Zafra, 2022).

Research into grit and self-regulation has revealed that grittier individuals participate in proactive behaviours to pre-emptively cultivate a constructive path of action for realising their goals, particularly when pursuing very ambitious long-term goals (Aspinwall and Taylor, 1997; Strauss et al., 2012; Sturman and Zappala-Piemme, 2017; Jordan et al., 2019a). Grit entails having a central superior goal and determinedly working towards it in the face of obstacles and setbacks, often for years or decades (Duckworth and Gross, 2014). As an agentic characteristic, grit is likely to facilitate one’s ability to adapt successfully to constantly changing work circumstances (Gregor et al., 2021). Grit also governs and regulates human behaviour (Hu et al., 2017; Min, 2018).

As a mediator, grit has been researched in relation to growth mindset, goal commitment and achievement outcomes (Tang et al., 2019) and personality, hardiness, resilience and subjective career success (Cunningham, 2018) among others. However, there is a dearth of research that focuses on grit in the career and employability context, particularly where grit is studied as the mediating variable. There are fewer studies that have been conducted within the South African workspace.

Han (2021) called for more experimental data to understand the mediating role of grit in individuals’ wellbeing. Tang et al. (2021) called for more studies to understand the resilience model of grit in other countries. Cunningham (2018) called for a more in-depth assessment of grit within the South African environment. This study responds to these calls for further research by addressing the following research question:

Does grit mediate the link between self-regulatory employability attributes and employability competency expectations?

The objective of the study was to explore grit as strengthening mechanism of the link between self-regulatory employability attributes (career agility, cultural ingenuity, proactive career resilience) and employer employability competency expectations (autonomy and leadership skills, and personal employability qualities). This association between the variables is likely to provide career counsellors and trainers with avenues to explore interventions to develop self-regulatory employability attributes that enhance grit which in turn may strengthen individuals’ employability competency.

This article is structured as follows: The variables of the study will first be explained, thereafter the method, measuring instruments, data analyses, results, discussion, practical implications, limitations and recommendation for future research are presented. Finally, a conclusion to the article is provided with the references utilised for this study.

## 2 Theoretical constructs

It is important to note that there are two conceptualisations of employability in this study. A self-regulatory conceptualisation that is reflected in the employability attributes (as antecedents), and a contextual conceptualisation that is reflected in self-perceived employability competency expectations as outcomes.

### 2.1 Self-regulatory employability attributes

Self-regulatory employability attributes is a psychosocial construct which refers to those career-related qualities that enable an individual to operate within a given (un) employment context (now and in the future) as an agentic, effective, efficient and healthy stakeholder (Van der Heijde, 2014; Coetzee, 2017).

The basic premise of self-regulatory employability attributes is that individuals who possess these attributes are agentic and internally motivated to self-develop, flourish and produce their own opportunities in their employment (Coetzee, 2019). Through self-directed behaviours, these individuals pivot their goal-directed activities and personal resources to achieving sustained employability over time and across changing circumstances (Sokol and Müller, 2007; Coetzee and Beukes, 2010; Van der Heijde, 2014; Nielsen, 2017; Coetzee, 2019).

This study is interested in examining the link between three self-regulatory employability attributes (career agility, cultural ingenuity and proactive career resilience), grit and perceptions of employer competency expectations. Research has indicated positive links between these three self-regulatory employability attributes and perceptions of employability (Coetzee, 2019) which may be explained by the psychological needs of self-regulated autonomy and competence from self-determination theory (SDT; Deci and Ryan, 2000). However, the link with grit is still unknown.

### 2.2 Self-regulated autonomy: career agility

When viewed from the employability context, self-regulated autonomy is the display of personal agency (autonomy) of an individual in overseeing their career and setting and executing goals that enhances one’s achievement of better person-environment consistency (Coetzee and Engelbrecht, 2020). Career agility alludes to self-regulated autonomy whereby individuals show agentic readiness to manage career goals, seek out new career development opportunities because of an openmindedness towards changing employment conditions (Coetzee, 2019, 2022). Research indicates the psychosocial mindset denoted by career agility as an important attribute for perceived employability competency (Potgieter et al., 2023).

### 2.3 Self-regulated competence: cultural ingenuity and proactive career resilience

Self-regulated competence is the tendency to influence the environment and achieve valuable outcomes within it (Deci and Ryan, 2000). Individuals perceive that they are capable and confident in their actions and behaviours that assist them in attaining specific career outcomes like employability and understanding the conditions that influence one’s career success and employability (Coetzee and Engelbrecht, 2020).

Cultural ingenuity reflects individuals’ skills and ingenuity in interacting with various groups of people within the culturally diverse employment and career context (Coetzee, 2019). Cultural ingenuity presupposes a sense of self-efficacy in initiating and nurturing relationships with individuals hailing from diverse cultural backgrounds, facilitating seamless intercultural communication, comprehending the customs, values and beliefs intrinsic to other cultures and astutely adapting to disparate social milieus (Abbe et al., 2007; Coetzee, 2019).

Proactive career resilience denotes the ability to confidently adapt to changes in the career environment, take advantage of opportunities to progress in the career and successfully action career plans despite challenges (Coetzee, 2019). Combined, career agility (the agentic readiness to embrace career self-management) and proactive career resilience (confidence in proactive career-related adaptation despite challenges) facilitate positive outlooks relating to the future, dealing with changes proactively as well as exhibiting self-initiative in seeking and recognising opportunities that advance the career-life (Chiaburu et al., 2006; van der Heijde and van der Heijden, 2006; Fugate and Kinicki, 2008; Coetzee, 2019).

### 2.4 Employability competency expectations

Employability competency expectations refer to those skills and qualities that employers expect current or prospective employees to have to be successful (Coetzee et al., 2019). This study focuses on autonomy and leadership skills and personal employability qualities as two employability competency expectations regarded important by employers and individuals’ confidence in gaining employment (Coetzee et al., 2019). Autonomy and leadership skills relate to the ability to function autonomously and exhibiting confidence in building networks, influence and persuade others and empower self and others. Personal employability qualities are important human capital resources such as the ability to manage and use time efficiently and productively, adapt to changing conditions, follow through and deliver on results and keeping knowledge and skills updated (Potgieter et al., 2023).

### 2.5 Grit

Grit denotes a non-cognitive, purpose-driven, context-specific goal-setting mindset that (1) influences individuals’ ability to establish and engage in purpose-driven long-term (higher order) goals by actively displaying perseverance and passion for such goals, while also (2) reflecting on the application of strategies for lower-order goals and adapting these strategies in the face of challenges or negative feedback (Duckworth, 2017; Datu et al., 2018; Jordan et al., 2019b; Datu et al., 2021; Schwepker and Good, 2022). Perseverance of effort and consistency of interest as compound state-like traits of grit have been found to be predictors of success, optimal functioning, performance and goal achievement (Duckworth et al., 2007; Duckworth and Quinn, 2009; Duckworth et al., 2011; West et al., 2016; Duckworth, 2017; Polirstok, 2017; Park et al., 2018). When faced with adversity the gritty individual responds by actively searching for alternative actions to pursue (Duckworth et al., 2007; Duckworth, 2017).

Grit has displayed significant positive impact across various settings, particularly higher goal achievement (Sheldon et al., 2015); academic achievement (Bowman et al., 2015); persistence in challenging tasks (Lucas et al., 2015) and remaining employed (Robertson-Kraft and Duckworth, 2014). Gritty individuals tend to display better adaptive psychosocial functions, such as psychological wellbeing (Disabato et al., 2016; Vainio and Daukantaitė, 2016; Datu et al., 2017); prosocial behaviour (Lan and Moscardino, 2019); healthy personal relationships (Lan, 2020), and less mental distress (Zhang et al., 2018). Furthermore, grit predicts the ability of a person, irrespective of internal facets such as genes and IQ, to devote to the required perseverance for both academic and professional success (Duckworth et al., 2010).

### 2.6 Integration: grit as mediating mechanism

Jin and Kim (2017) found that grit is strongly related to both the self-regulatory autonomy and competence needs of employability. The employability attribute of career agility involves putting in the effort to engage in lifelong learning, persevering in keeping up to date with new job or career requirements, ensuring that one remains interested through setting stimulating goals and arriving at creative solutions all the while persevering despite challenges of change (Coetzee, 2019).

The employability attribute of cultural ingenuity is related to persevering to learn more about different cultures and how to communicate and work with people from diverse backgrounds. Being culturally ingenious involves the ability of people to initiate and maintain relationships with people from multicultural backgrounds and to enjoy the process (that is, being interested in it; Coetzee, 2019). Once again, cultural ingenuity relates to both aspects of grit (perseverance of effort and consistency of interest).

Proactive career resilience denotes an inherent perseverance of effort attribute of grit. The self-regulatory confidence in proactively adapting to and capitalising on new career development opportunities because of changing employment conditions may foster the intrinsic motivation to persevere and remain consistent in one’s interest for employability (Duckworth et al., 2007; Perkins-Gough, 2013; Duckworth, 2017; Stoffel and Cain, 2018).

Research on the mediating role of grit in the employability context seems non-existent, especially regarding the link between self-regulatory employability attributes and employability competency. Grit as mediating mechanism may potentially deepen understanding of the psychological processes that link self-regulated attributes of employability and confidence in employability competency. Grit describes the unrelenting commitment towards completion of a particular task, for example achieving employability competency, despite failures, setbacks, and adversity (Duckworth et al., 2007). Grit is malleable and can be developed and improved, based on the amount of interest invested as people age, understand their life purpose and even as they develop passion and perseverance (Vela et al., 2015; Hill et al., 2016; Datu, 2017; Duckworth, 2017). In essence, when employability is viewed as the long-term career goal, gritty individuals will strive to upskill or reskill their employability attributes in the hopes of meeting employer employability competency expectations. According to Salisu et al. (2020), those with high levels of grit are more likely to capably employ their competencies better since they are less concerned with short-term goals and less affected by the shocks, obstacles or disappointments in their surrounding environments because of efforts to meet the long-term goal.

In relation to this study then, it can be assumed that individuals who seek to enhance their career agility, cultural ingenuity and proactive career resilience (self-regulatory employability attributes) would also be more likely to meet the employability competency expectations of autonomy/leadership and personal employability qualities through their grit. When individuals engage in setting career and employability goals, they direct their attention and energies into meeting those goals (Locke and Latham, 2002). The more challenging the goals, the more individuals will persevere and stick to their goals (Locke and Latham, 2002). It is possible then that when meeting employers’ employability competency expectations is viewed as the goal, the self-regulatory development of employability attributes (career agility, cultural ingenuity, proactive career resilience) will enhance individuals’ perseverance of effort and consistency of interest (grit) to meet the goal of employability competency. This will be empirically tested as follows:

*H*1: High levels of employability attributes are positively associated with employability competency expectations through individuals’ grit.

## 3 Methods

### 3.1 Sample

Primary data was obtained from a sample (*N* = 308) of South African adults (18 years and older) from various sectors. The sample was represented by predominantly employed (85%) females (59%). Indians (64%) made up the majority of the sample, followed by African participants (16%), White persons (16%) and mixed-race (s) participants (4%).

### 3.2 Procedure

The data were electronically collected through an online survey platform after obtaining ethical clearance from the University of South Africa Research Ethics Committee. Informed consent was also obtained from the participants. Respondents’ rights to confidentiality, voluntary participation and anonymity were upheld throughout the process.

### 3.3 Measuring instruments

Respondents completed the Employability Attributes Scale (EAS 4.0, Coetzee, 2019); the Employability Competency Inventory (ECI; Coetzee et al., 2019) and the Short Grit Scale (SGS; Duckworth and Quinn, 2009). They also provided data on their demographics.

#### 3.3.1 Employability attributes scale

Career agility (13 items), cultural ingenuity (7 items) and proactive career resilience (8 items) were measured using the employability attributes scale (EAS 4.0; Coetzee, 2019). Examples of items include, “I am generally willing to consider new ideas” (career agility), “I understand the values and beliefs of other cultures” (cultural ingenuity), “I anticipate and take advantage of changes in my career environment (proactive career resilience).” Responses were measured on a 7-point Likert scale: 1 = definitely disagree; 7 = strongly agree. The Cronbach’s alpha coefficient for the three sub-scales was 0.94. The overall scale obtained a Cronbach’s alpha of 0.98. Ismail (2023) provided evidence of construct validity of the scale.

#### 3.3.2 Employability competency inventory

Autonomy/leadership skills (6 items) and personal employability qualities (8 items) were measured using the employability competency inventory (ECI; Coetzee et al., 2019). Examples of items include, “Ability to empower self and others” (autonomy), and “Ability to work under pressure” (personal employability qualities). Responses were measured on a 5-point Likert scale: 1 = I do not feel confident at all; 5 = I feel highly confident. The Cronbach’s alpha coefficient for the two sub-scales was 0.95 (autonomy) and 0.94 (personal employability qualities). The overall scale obtained a Cronbach’s alpha of 0.98. Ismail (2023) provided evidence of the construct validity of the scale.

#### 3.3.3 Short grit scale

Consistency of interest (4 items) and perseverance of effort (4 items) were measured using the 8-item short grit scale (SGS; Duckworth and Quinn, 2009). Examples of items include, “I often set a goal but later choose to pursue a different one” (Consistency of Interest), and “I finish whatever I begin” (Perseverance of Effort). Responses were measured on a 5-point Likert scale: 1 = not like me at all; 5 = very much like me. The Cronbach’s alpha coefficient for the two-subscale was 0.84 (consistency of interest) and 0.72 (perseverance of effort). The overall scale obtained a Cronbach’s alpha of 0.79. Ismail (2023) provided evidence of the construct validity of the scale.

## 4 Data analyses

Simple mediation analysis was conducted in order to investigate whether the mediating effect of individuals’ grit between employability attributes (independent variables) and employability competency expectations (dependent variables) is significant.

The JASP (2022) computer software package was used to perform the mediation analysis with Delta method standard errors and maximum likelihood estimator. Following the guidelines of Hayes (2013), significance mediation (indirect) effects were established at the more stringent 95% bias-corrected bootstrapped lower and upper-level confidence intervals. Significant mediating effects were evident when the lower-level confidence interval (LLCI) and upper-level confidence interval range did not contain zero (that is, the interval values fall either above or below zero). Research indicates bias-corrected confidence intervals to have the highest statistical power when analysing mediation effects (Hayes and Scharkow, 2013).

Following the guidelines of MacKinnon (2008), simple mediation modelling was applied to first test whether the exogenous or independent variables (employability attributes) had a direct effect on the endogenous (or dependent) variable (employability competency expectations). Second, the respective pathways from the two exogenous variables via the mediator variable (grit) to the endogenous variable and its sub facets were tested for significant indirect (mediating) effects.

The next step was to examine indirect (mediating) effects of sub facets of these constructs. Structural equation modelling was employed to test various combinations of indirect effects via parallel mediation. This approach helped to control for other potential mediators in the model and to obtain a deeper understanding of the psycho-social process through which the employability attributes through the grit variables relate to employer employability competency expectations (Hayes and Scharkow, 2013; Kane and Ashbaugh, 2017).

The JASP (2022) computer software package and SAS (CALIS Procedure) software version 9.4 (SAS, 2013) with maximum likelihood estimator were used to perform mediation analyses via structural equation modelling. In the parallel mediation models, the mediators were allowed to correlate but not to influence each other. Parallel mediation can test indirect effects of each proposed mediator while accounting for the shared variance between them (Kane and Ashbaugh, 2017).

## 5 Results

### 5.1 Descriptive statistics and bi-variate correlations

In Table 1, the internal consistency reliability (Cronbach α), composite reliability (CR), means, standard deviations and correlations between the constructs are provided. Table 1 shows that the construct scales had acceptable (0.69) to high (0.94) internal consistency reliability. Employability attributes correlated positively with all the employability competency expectations (0.60 ≤ *r* ≤ 0.72; *p* ≤ 0.001; large practical effect). Employability competency expectations correlated positively with all the grit variables (0.26 ≤ *r* ≤ 0.60; *p* ≤ 0.001; moderate to large practical effect). Employability attributes correlated positively with all the grit variables (0.14 ≤ *r* ≤ 0.63; *p* ≤ 0.05; small to large practical effect). These results indicated significant positive associations between the employability attributes, employability competency expectations and the grit variables. The highest correlations were observed between overall EAS and overall ECI (*r* = 0.78, *p* ≤ 0.05; large practical effect). All correlations between the scales were below <0.80 thereby minimising potential multicollinearity concerns.

**Table 1:** Means, standard deviations, reliabilities, and correlations among study variables.

Variables	Composite Reliability (CR)	Cronbach Alphas	Mean	Standard Deviation	Personal employability qualities	Autonomy/ leadership	Overall ECI	Career agility	Cultural ingenuity	Proactive career resilience	Overall EAS	Consistency of Interest	Perseverance of Effort	Overall SGS
Personal employability qualities	0.93	0.94	3.92	0.89	-									
Autonomy/ Leadership	0.92	0.95	3.81	0.97	0.83 ***	-								
Overall ECI	0.97	0.98	3.87	0.81	0.93 ***	0.90 ***	-							
Career agility	0.96	0.94	5.70	1.12	0.71 ***	0.70 ***	0.74 ***	-						
Cultural ingenuity	0.93	0.94	5.52	1.18	0.60 ***	0.62 ***	0.64 ***	0.68 ***	-					
Proactive career resilience	0.94	0.94	5.42	1.14	0.72 ***	0.72 ***	0.75 ***	0.81 ***	0.74 ***	-				
Overall EAS	0.98	0.98	5.47	1.03	0.75 ***	0.75 ***	0.78 ***	0.90 ***	0.84 ***	0.93 ***	-			
Consistency of Interest	0.81	0.84	3.20	0.90	0.28 ***	0.26 ***	0.26 ***	0.20 ***	0.14 *	0.22 ***	0.22 ***	-		
Perseverance of Effort	0.69	0.72	4.03	0.65	0.60 ***	0.55 ***	0.57 ***	0.58 ***	0.44 ***	0.63 ***	0.63 ***	0.32 ***	-	
Overall SGS	0.88	0.79	3.62	0.63	0.49 ***	0.45 ***	0.46 ***	0.42 ***	0.31 ***	0.46 ***	0.45 ***	0.88 ***	0.70 ***	-

*N* = 308; ***p ≤ 0.0001; *p ≤ 0.05.

### 5.2 Simple mediation analysis

Table 2 displays the standardised total, direct and indirect effects of the SEM model. Table 2 shows that employability attributes (*β* = 0.36; LLCI = 0.28; ULCI = 0.43) had significant and positive direct effects on employability competency expectations. In terms of indirect effects, Table 2 further shows that employability attributes through grit had a significant and positive indirect effect (*β* = 0.03; LLCI = 0.01; ULCI = 0.05) on employability competency expectations. The total effect of employability attributes (*β* = 0.39; LLCI = 0.31; ULCI = 0.47) on employer employability competency expectations was also significant, suggesting only a partial mediation effect. The total indirect effects for employability attributes (*β* = 0.04; LLCI = 0.01; ULCI = 0.06) were also significant.

**Table 2:** Results of mediation analysis: employability competency expectations as overall dependent variable.

Direct effects on employer employability competency expectations						
Variable	β	SE	z	*p*	LCI	UCI
Employability attributes	0.36	0.04	8.79	0.00	0.28	0.43
Indirect effects on employer employability competency expectations						
Variable	β	SE	z	*p*	LCI	UCI
Employability attributes through grit	0.03	0.01	2.12	0.02	0.01	0.05
Total effects on employer employability competency expectations						
Variable	β	SE	z	*p*	LCI	UCI
Employability attributes	0.39	0.04	9.76	0.00	0.31	0.47
Total indirect effects on employer employability competency expectations						
Variable	β	SE	z	*p*	LCI	UCI
Employability attributes	0.04	0.01	2.78	0.01	0.01	0.06

*N* = 308.

### 5.3 Structural equation modelling

**Figure 1:**
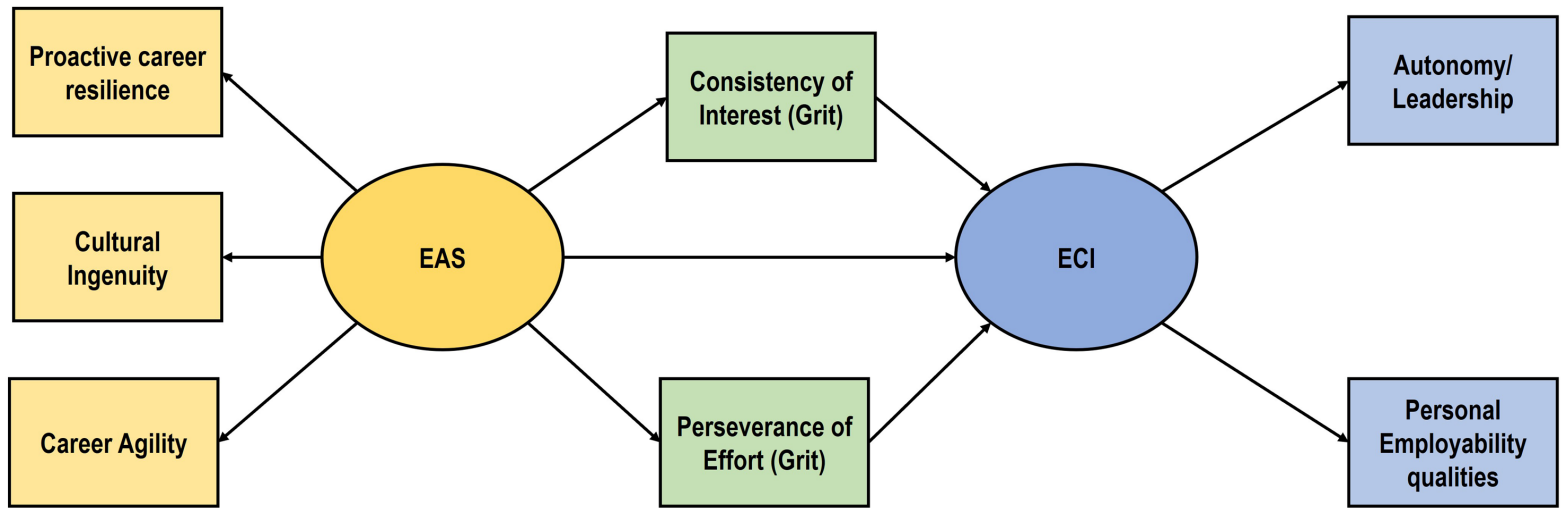
Parallel mediation model. Source: Ismail, 2023.

Proactive career resilience, cultural ingenuity and career agility were loaded as subfactors onto the overall employability attributes factor (exogenous variable). Autonomy/leadership and personal employability qualities were loaded as two subfactors onto the overall construct of employability competency expectations as endogenous variable. The two sub facets of grit (consistency of interest and perseverance of effort) acted as parallel mediators. This is illustrated in Figure 1.

**Figure 2:**
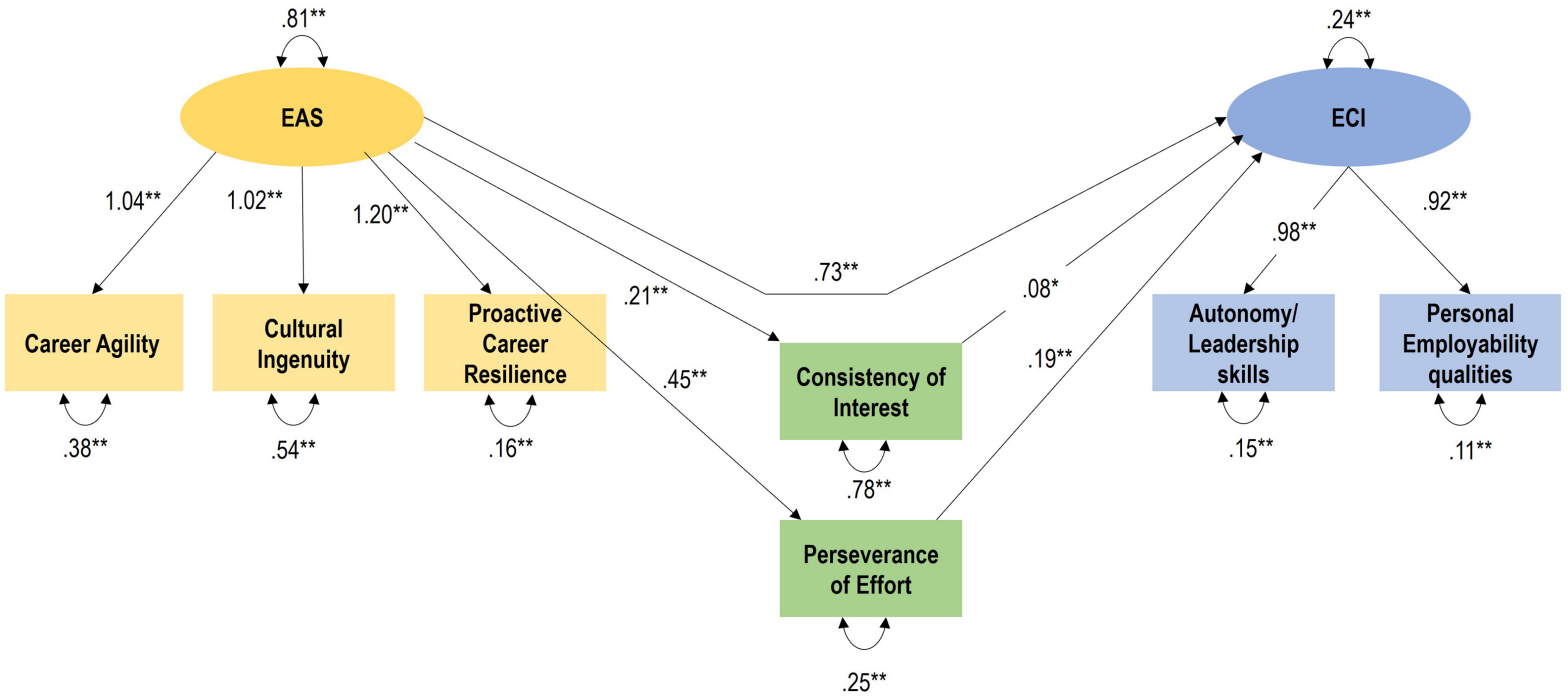
Structural model of mediation model.

Table 3 provides the model fit statistics of the mediation model. The following rules of thumb (threshold values) were applied for good model fit (Hair et al., 2019): chi-square/df ≤ 3; RMSEA ≤0.06 or ≤ 0.08; SRMR ≤0.05; CFI ≥ 0.90. The model had a good fit with the data: chi-square/df = 3.99; CFI = 0.98; RMSEA = 0.06, SRMR = 0.04. The path regressions for the model are provided in Table 4. The structural model of the mediation model is provided in Figure 2. Overall, the empirical results provided support for the research hypothesis (H1).

**Table 3:** Path regression coefficients: mediation model.

Latent variables	Path	Manifest variables	Estimate	Standard error	*t* value
EAS	*→*	Cultural Ingenuity	0.78	0.03	31.20***
EAS	*→*	Career agility	0.84	0.02	40.71***
EAS	*→*	Proactive Career Resilience	0.94	0.01	71.07***
ECI	*→*	Autonomy	0.92	0.02	60.56***
ECI	*→*	Personal Employability qualities	0.93	0.01	64.52***
ECI	*←*	Consistency of Interest	0.08	0.04	2.10*
ECI	*←*	Perseverance of Effort	0.14	0.05	2.78*
ECI	*←*	EAS	0.73	0.05	16.20***
Consistency of Interest	*←*	EAS	0.20	0.06	3.58***
Perseverance of Effort	*←*	EAS	0.63	0.04	16.83***

*N* = 308; ***p ≤ 0.0001; *p ≤ 0.05.

**Table 4:** Structural equation modelling: direct and indirect effects (SAS calis procedure).

Direct effects				
Path		Estimate (β)	Std error	*t* value
Autonomy/leadership	*←* ECI	0.98	0.02	42.83***
Personal employability qualities	*←* ECI	0.92	0.02	41.11***
Career agility	*←* EAS	1.04	0.04	25.96***
Cultural ingenuity	*←* EAS	1.02	0.05	22.44***
Proactive career resilience	*←* EAS	1.20	0.03	35.59***
Consistency of Interest	*←* EAS	0.21	0.06	3.52***
Perseverance of Effort	*←* EAS	0.45	0.03	13.47***
ECI	*←* Consistency of Interest	0.08	0.04	2.10*
ECI	*←* Perseverance of Effort	0.19	0.07	2.76**
ECI	*←* EAS	0.73	0.04	16.87***
Indirect effects				
Autonomy/leadership	*←* Consistency of Interest	0.08	0.04	2.11*
Autonomy/leadership	*←* Perseverance of Effort	0.19	0.07	2.78**
Autonomy/leadership	*←* EAS	0.82	0.04	19.59***
Personal Employability qualities	*←* Consistency of Interest	0.07	0.04	2.11*
Personal Employability qualities	*←* Perseverance of Effort	0.18	0.06	2.78**
Personal Employability qualities	*←* EAS	0.77	0.04	20.21***
ECI	*←* EAS	0.10	0.03	3.11**

*N* = 308; ***p ≤ 0.0001; **p ≤ 0.01; *p ≤ 0.05.

## 6 Discussion

This purpose of this study was to explore whether grit mediates the relationship between self-regulatory employability attributes and employability competency expectations. Although self-regulatory employability attributes were positively associated with employability competency expectations, this link was further (although not strongly) strengthened through grit.

The present findings suggest that the self-regulatory qualities of career agility (agency in managing career goals, seeking out new career development opportunities and being openminded towards changing conditions; Coetzee et al., 2019; Coetzee, 2022), cultural ingenuity (insight into own and others’ values and beliefs, and confidence in communicating and engaging with other cultures: Coetzee, 2019) and proactive career resilience (self-efficacious adaptation while capitalising on change for career advancement despite challenges (Han et al., 2021; Peeters et al., 2022) strengthen grit. In turn, grit seems to heighten confidence in one’s ability to function autonomously and exhibiting leadership skills (i.e., take leadership in building networks, influence and persuade others, and empower self and others (Coetzee et al., 2019). Grit also appears to strengthen confidence in one’s personal employability qualities (i.e., ability to stay relevant by updating one’s knowledge and skills, adapting to changing work conditions and pressure while also following through and delivering results; Coetzee et al., 2019).

The strong and positive direct association between the three self-regulatory employability attributes and individuals’ confidence in their employability competency (autonomy and leadership, and personal employability qualities) could be attributed to the agentic and internally motivated drive to self-develop, flourish and produce personal employment opportunities that underpin self-regulatory employability attributes (Sokol and Müller, 2007; Van der Heijde, 2014; Nielsen, 2017; Coetzee, 2019). In this regard, the self-regulated employability attributes seem to denote key psychosocial mindsets that activate especially perseverance of effort (heightened motivation to actively persevere in behavioural effort: Datu (2021), and to a lesser degree consistency of effort persistent interest in goal achievement: Datu (2021), which in turn, help strengthen confidence in employability competency.

The finding corroborates empirical evidence that grit as a malleable compound state-like trait, is linked to agentic, behavioural, cognitive and emotional engagement, autonomous motivation, and mastery- and performance-approach goals (Chen et al., 2018; Datu et al., 2018). The stronger mediating role of perseverance of effort in relation to consistency of effort is in agreement with arguments that a lower level of consistency of effort points to individuals’ capacity for self-variability, that is, a tendency to calibrate one’s goals, interests and behaviour based on situational demands or cues in achieving long-term aspirations (Datu, 2021). Individuals with high perseverance of effort and low consistency of effort generally exhibit significantly higher levels of hope and lower anxiety (Datu and Fong, 2018).

Overall, the findings support the view that grit as a protective resilience factor help individuals to persevere and stay appropriately consistent in their interest to meet employer employability competency expectations to enable them to realise employability goals. Previous research corroborates the role of grit in remaining employed (Robertson-Kraft and Duckworth, 2014) and staying focused on realising long-term goals of employability despite adversity or obstacles (Salisu et al., 2020). In this study, the self-regulated autonomy and competence attributes of career agility, cultural ingenuity and proactive resilience (employability attributes) gave impetus to participants’ drive to persevere and stay relatively consistent in their efforts (grit) to meet the employability competencies of autonomy and leadership, and the personal employability qualities required by employers. Tang et al. (2021) explain grit as a compensatory and protective resilience factor in realising goals of employability. When facing challenges in their goal pursuit, less gritty individuals were more likely to give up on their goal (Yu et al., 2021). This occurs when individuals have fewer skills (employability attributes) and perceive themselves as being unable to meet their goal (meeting employability competency expectations; Hembree, 1990; Ashcraft and Kirk, 2001; Yu et al., 2021).

## 7 Practical implications

This study revealed important preliminary insights that extended research on grit. The findings suggest that self-regulatory employability attributes (cultural ingenuity, proactive career resilience and career agility) and grit (perseverance of effort and consistency of interest) should be developed and strengthened to enhance the employability competency expectations of autonomy/leadership skills and personal employability qualities. This can be done through educational learning as well as through training in the workspace. The link between employability attributes and grit is undeniably lifelong learning and adapting to the changes one is faced with in changing employment environments (Chen and Hong, 2020a,b; Nilforooshan, 2020; Öztemel and Akyol, 2021). If individuals continue to persevere in enhancing their employability attributes, they provide themselves with an added advantage of meeting employability competency expectations of current and prospective employers. Through adapting to changing conditions, they position themselves as the talent that employers seek.

Not only is adapting to the changes and challenges a means to secure employment, but within the digital era work world, adapting, positions individuals in a positive and strategic mind space wherein they view change and challenge as a way to learn and grow. This means that they will eagerly seek out new ways to engage with technology and their careers, thereby making them productive assets to any organisation and within any workspace they find themselves in.

It is important to understand that the role of the surrounding environment in supporting this process should not be excluded. The social environment of the organisation is generally considered instrumental to meaningful career goal achievement (Xie et al., 2017). Employers, families, friends, colleagues, educational systems and governments should provide the resources and support to individuals to assist them in meeting these objectives (Tang et al., 2019; Tewell, 2020). Positive associations between individuals’ career agility, for example, and the organisation’s appraisal as fulfilling its obligation in providing supportive conditions enable adaptation learning and upskilling in today’s work world (Coetzee, 2022).

This study further raises awareness among employers and organisations on the areas of training and development that need to be addressed. Career counsellors and human resource development practitioners should utilise the findings of this study to design interventions that will develop individuals’ employability attributes and grit. Individuals can also make use of these results, to proactively engage in exercises and programmes to build their self-regulatory employability and grit by enrolling in online courses or researching ways in which they can upskill. This will in turn assist individuals in meeting employer employability competency expectations thereby enhancing their employability.

## 8 Limitations and recommendations for future research

The current study was conducted in South Africa and due to contextual differences, the small sample size and the fact that the sample comprised mainly Indian South Africans, mean that the findings cannot be generalised. Few studies have focused on the role of grit in enhancing employability, particularly within the South African context. Future studies can replicate this study in different countries and setting and with a more representative sample.

The cross-sectional design of this study limits any causal inferences. It is recommended that longitudinal studies are conducted to examine the true causal change in employer employability competency expectations as a result of grit as intervening mechanism. Longitudinal research can further assess the malleability of grit and grit profiles of individuals (e.g., high or low perseverance of effort versus high or low consistency of effort) over time in relation to their self-regulatory employability attributes and employability competency.

Theoretically, grit is not a self-perception, nor is it directly observable (Duckworth and Yeager, 2015). To adequately measure one’s passion and perseverance would require both first-person and third-person (Usher et al., 2019). Here, we simply measured individuals’ self-perceived grit. Future research can include the third-person perspective as well.

## 9 Conclusion

The results of this study revealed the influence of grit in strengthening the link between employability attributes (particularly career agility, cultural ingenuity and proactive career resilience) and employability competency expectations (particularly autonomy/leadership skills and personal employability qualities). The findings underscore the need for continuous skill development, resilience, and holistic career approaches to meet the evolving demands of the contemporary world of work. Career interventions, educational learning and training should consider structuring meeting employability competency expectations as a goal that needs to be achieved through fostering grit. Through building career agility, cultural ingenuity and proactive career resilience while also strengthening grit, individuals should be successful in meeting their goal in enhancing their employability in the contemporary world of work.

## Data availability statement

The raw data supporting the conclusions of this article will be made available by the authors, without undue reservation.

## Ethics statement

The studies involving humans were approved by UNISA College of Economic and Management Sciences Research Ethics Review Committee. The studies were conducted in accordance with the local legislation and institutional requirements. The participants provided their written informed consent to participate in this study.

## Author contributions

SI: Writing – original draft. IP: Writing – review & editing. MC: Writing – review & editing.
